# ACURATE neo: How Is This TAVR Valve Doing to Fit into an Increasingly Crowded Field?

**DOI:** 10.1007/s11886-020-01364-4

**Published:** 2020-08-08

**Authors:** Taishi Okuno, Jonas Lanz, Thomas Pilgrim

**Affiliations:** Department of Cardiology, Inselspital, Bern University Hospital, University of Bern, CH-3010 Bern, Switzerland

**Keywords:** Transcatheter aortic valve replacement, ACURATE neo, Self-expanding, Balloon-expandable, Transcatheter heart valve, SCOPE

## Abstract

**Purpose of Review:**

Critical appraisal of the available evidence on the self-expanding ACURATE neo transcatheter heart valve (THV) for the treatment of aortic valve disease.

**Recent Findings:**

In an investigator-initiated, multicenter, randomized non-inferiority trial with broad inclusion criteria, ACURATE neo failed to meet non-inferiority compared with SAPIEN 3 with regard to a primary composite safety and efficacy endpoint at 30 days. The difference was driven by higher rates of moderate or severe paravalvular regurgitation and higher rates of acute kidney injury. In turn, registry data suggest that the safety and efficacy profile of the ACURATE neo is comparable to that of other commercially available devices. Randomized evidence indicated favorable hemodynamic results with large effective orifice areas and low residual gradients.

**Summary:**

The self-expanding ACURATE neo THV is associated with higher rates of residual aortic regurgitation compared to the balloon-expandable SAPIEN 3 THV. The supra-annular design with low residual gradients may be advantageous in patients with small anatomy and mild degree of calcification.

## Introduction

Since the introduction of transcatheter aortic valve replacement (TAVR) as an alternative for inoperable patients with severe aortic stenosis [1], advances in treatment strategies and refinement of valve systems have expedited the expansion of TAVR to surgical low-risk patients [2, 3]. Nowadays, TAVR is considered a valuable treatment strategy across the entire spectrum of surgical risk based on robust evidence derived from iterative randomized clinical trials and large-scale real-world registries [4]. Strategy trials comparing TAVR to surgical aortic valve replacement (SAVR) have been performed with different generations of transcatheter heart valve (THV) systems of the balloon-expandable SAPIEN series (Edwards Lifesciences, Irvine, CA, USA) and the self-expanding CoreValve/Evolut series (Medtronic, Minneapolis, MN, USA). A number of novel THVs have been introduced with the aim to overcome limitations of these two dominant market players [5••, 6–9]. Among them, the ACURATE neo THV (Boston Scientific, Marlborough, MA, USA) is one of the leading devices, which has a unique valve design and deployment mechanism [5••]. In this review, we summarize the available evidence on this device and elucidate its strengths and weaknesses as compared to other commercially available THV devices.

## The ACURATE neo: Device Characteristics and Deployment Mechanism

**Fig. 1:**
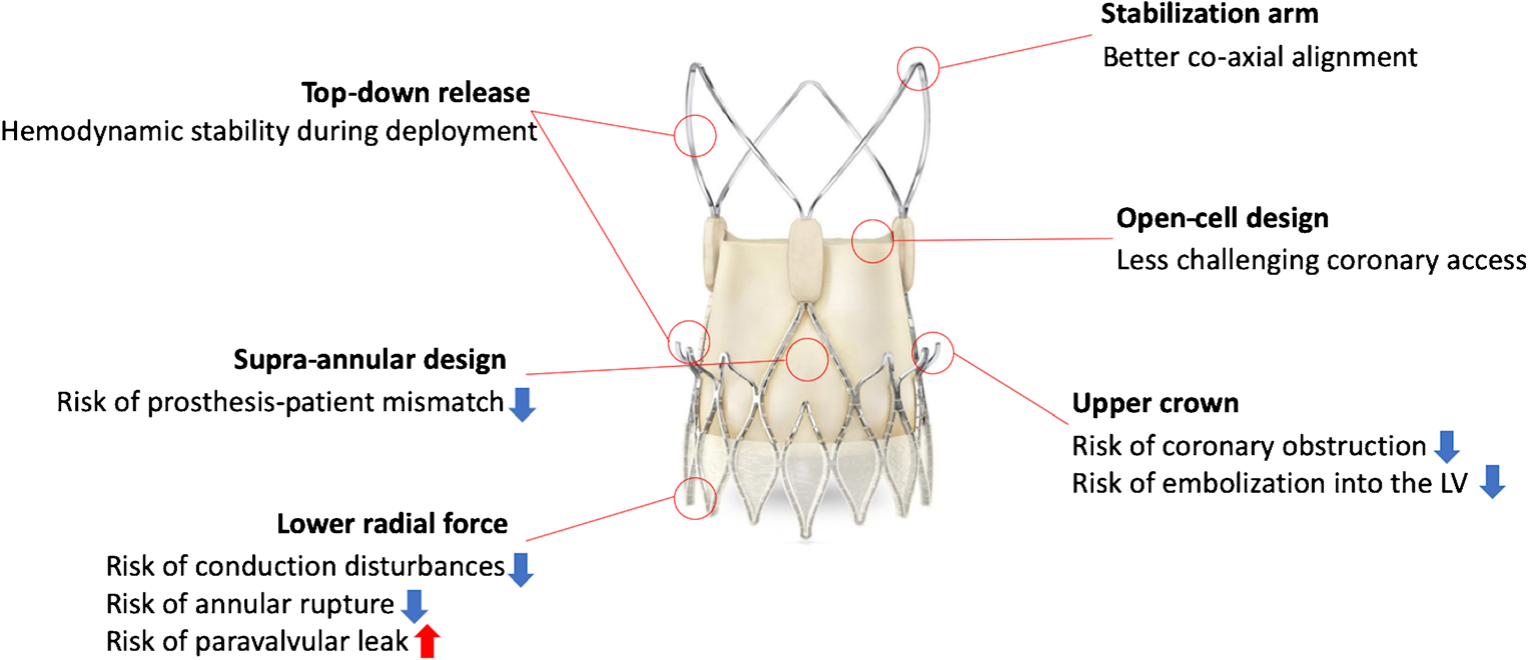
Unique design of the ACURATE neo transcatheter heart valve. Overview of the unique features of the device and their clinical relevance are presented. (Image/content provided courtesy of Boston Scientific. © 2020 Boston Scientific Corporation or its affiliates. All rights reserved)

The ACURATE neo is composed of porcine pericardial leaflets mounted on a self-expanding nitinol stent frame in supra-annular position and is implanted in a top-down two-step release mechanism. This unique deployment mechanism minimizes peri-procedural outflow obstruction and allows for stable positioning without rapid ventricular stimulation. Three stabilization arches and the protruding upper crown further enhance co-axial deployment and device stabilization (Fig. 1). In addition, the upper crown may deter the native leaflets from the coronary ostia and reduce the risk of coronary obstruction (Fig. 1). Currently, three sizes of the THV (S, M, L) are available and accommodate aortic annular diameters ranging from 21 to 27 mm. The delivery system is compatible with an 18-Fr (15-Fr expandable) sheath. Pre-dilatation is required prior to valve implantation according to the instructions for use in order to allow adequate device expansion.

## The ACURATE neo Device for Native Aortic Valve Stenosis

Several studies have investigated the clinical outcomes up to 1 year after implantation of the ACURATE neo THV for treatment of severe aortic stenosis in elderly patients (Table 1) [10–12, 13•, 14•, 15, 16, 17•, 18•, 19, 20]. These studies have proposed some strengths and drawbacks of ACURATE neo, which may guide how to optimally tailor the device to the most appropriate anatomy.

**Table 1:** Results of single-arm studies

Study cohort Author (year)		Baseline			Procedural outcomes					Echocardiographic outcomes			Mortality	
		*N*	Age	Surgical score	Annular rupture	Device embolization/dislocation	Coronary obstruction	Major vascular complication	Permanent pacemaker	EOA (cm^2^)	Mean PG (mmHg)	PVL	30-day mortality	1-year mortality
*CE-approval cohort+* Möllman et al. (2017) [10]		89	84 ± 4	ES I: 26.5 ± 7.7%	1 (1.1%)	2 (2.2%)	0 (0%)	3 (3.4%)	8 (10.3%)	1.8 ± 0.3	8.0 ± 2.9	3 (4.5%)	3 (3.4%)	20 (22.5%)
Hamm et al. (2017)*^1^ [11]		219	81 ± 4	LES: 19.3 ± 13.9%	0 (0%)	1 (0.5%)	0 (0%)	11 (5.0%)	27 (12.3%)	NA	10.6 ± 9.2	4 (1.9%)	7 (3.2%)	5.2%
Toggweiler et al. (2018)*^2^ [12]		175	83 ± 6	STS: 4.1 ± 2.4%	NA	NA	NA	12 (6.9%)	4 (2.3%)	2.0 ± 0.4	6.9 ± 3.7	8 (4.6%)	1 (0.6%)	NA
*SAVI TF registry* Möllman et al. (2018) [13•] Kim et al. (2018) [14•]		1000	81 ± 5	ES II: 6.6 ± 7.5% STS: 6.0 ± 5.6%	0 (0%)	NA	0 (0%)	32 (3.2%)	83 (8.3%)	1.8 ± 0.5	8.4 ± 4.0	35 (4.1%)	14 (1.4%)	78 (8.0%)
Kim et al. (2018) [15]		500	82 (79–85)	LES: 18.3% (11.9–26.6) STS: 4.4% (3.1–6.6)	0 (0%)	6 (1.2%)	0 (0%)	46 (9.2%)	51 (10.2%)	1.6 (1.4–1.9)	8.0 (6.0–11.0)	24 (4.8%)	16 (3.3%)	NA
Pellegrini al. (2020) [16]		151	81 ± 6	ES II: 5.1% (2.9–7.1)	NA	NA	NA	21 (13.9%)	16 (10.6%)	NA	7.8 ± 3.3	2 (1.4%)	1 (0.7%)	5 (3.3%)
*Bicuspid aortic valve*														
Mangieri et al. (2018) [17•]	Tricuspid	658	82 ± 4	STS: 5.1 ± 3.6% LES: 20.1 ± 13.4%	0 (0%)	NA	1 (0.1%)	55 (8.3%)	57 (8.6%)	NA	8.4 ± 4.2	9 (3.1%)	17 (2.8%)	NA
	Bicuspid	54	80 ± 5	STS: 4.7 ± 2.7% LES: 17.7 ± 10.7%	0 (0%)	NA	1 (1.8%)	6 (11.1%)	5 (9.2%)	NA	9.8 ± 4.2	4 (7.4%)	2 (3.7%)	NA
*Valve-in-valve*														
Holzamer et al. (2019) [18•]		85	77 ± 8	STS: 6.8 ± 6.0% ES II: 11.4 ± 7.9%	0 (0%)	2 (2.4%)	1 (1.2%)	4 (4.7%)	1 (1.2%)	1.5 ± 0.4	16 ± 8	1 (1.2%)	4 (4.7%)	NA
*Pure aortic regurgitation*														
Toggweiler et al. (2018) [19]		20	79 ± 8	STS: 8.3 ± 9.3%	NA	0 (0%)	NA	1 (5%)	3 (15%)	2.2 ± 0.6	6 ± 3	1 (5%)	0 (0%)	NA
Purita et al. (2019) [20]		24	79.4 (50–88)	ES II: 5 ± 4.05 STS: 3.9 ± 2.37	0 (0%)	2 (8.3%)	0 (0%)	1 (4.1%)	4 (21.1%)	1.9 (1.2–2.2)	6.5 (4–16)	2 (8.3%)	1 (4.1%)	2 (11.7%)

*EOA* effective orifice area, *PG* pressure gradient, *PVL* paravalvular leak, *ES I* = Euroscore I, *ES II* Euroscore II, *LES* logistic Euroscore, *STS* Society of Thoracic Surgeons predicted risk of mortality, *NA* not available/assessed

+ Clinical events were adjudicated according to the VARC-2 criteria (no other studies comprised independent event adjudication or core laboratory echocardiographic assessment)

*^1^Ninety-nine patients were treated with transapical ACURATE TA

*^2^Minimizing trauma strategy: pre-dilation with 1–3 mm smaller balloon than perimeter-derived annular diameter, post-dilation with 1–2 mm smaller balloon than perimeter-derived diameter only in case of relevant aortic regurgitation or increased mean gradient > 10 mmHg

The top-down two-step deployment mechanism with stabilization arches and an upper crown anchoring the device in the aortic annulus facilitates stable deployment and mitigates the risk of device migration and potentially also coronary obstruction (Fig. 1). Malpositioning occurred in 0.5 to 2.2% across studies conducted in patients treated for severe stenosis of the native aortic valve [10, 11, 15]. The incidence of coronary obstruction was negligible when the ACURATE neo device was implanted in native tricuspid aortic valves [10, 11, 13•, 15]. The nitinol frame of the ACURATE neo THV has a lower radial force as compared to other self-expanding THVs (Fig. 1), thus requiring pre-dilatation and increasing the need for post-dilatation [5••]. The lower radial force may lead to less mechanical injury to the conduction system (Fig. 1) and explain the relatively low need for a new permanent pacemaker ranging from 8.3 to 12.3% [10–12, 13•, 15, 16, 17•], which is considerably lower than reported pacemaker rates of other self-expanding THVs (17.4 to 34.1%) [3, 21]. Evidence from a small multicenter study suggested that the rate of new permanent pacemakers can be reduced to 2.3% when applying a minimizing trauma strategy using a smaller balloon for pre- and post-dilation [12]. On the downside, the lower radial force may translate into malappositioning of the device to the aortic annulus and increase the risk of paravalvular regurgitation despite the presence of an outer skirt (Fig. 1). In a randomized trial among 739 patients from 20 centers, the occurrence of valve-related dysfunction, mainly moderate or severe aortic regurgitation, at 30 days, was significantly higher among patients treated with ACURATE neo (9%) compared to Edwards SAPIEN 3 (3%) (*P* < 0.0001) [5••]. In contrast, site-reported incidence of relevant paravalvular regurgitation (moderate to severe) in observational studies was considerably lower, ranging from 1.4 to 4.8% [10–12, 13•, 14•, 15, 16]. The supra-annular design of the THV achieves a larger effective orifice area (EOA) with lower transprosthetic gradients, and potentially prevents prosthesis-patient mismatch (Fig. 1) [22]. Across the studies, EOA and mean transprosthetic gradients ranged from 1.6 to 2.0 cm^2^ and from 6.9 to 10.6 mmHg (mean or median), respectively [10–12, 13•, 14•, 15, 16, 17•]. In a propensity-score-matched comparison in patients with a small aortic annulus (area < 400 mm^2^), the ACURATE neo THV was associated with a significantly lower incidence of prosthesis-patient mismatch as compared with the SAPIEN 3 THV [23]. Finally, 30-day mortality was reported as 0.6 to 3.4%, which were comparable to the results of TAVR randomized clinical trials [10–12, 13•, 14•, 15, 16, 17•].

## The ACURATE neo for Bicuspid Aortic Valve

Bicuspid anatomy was an exclusion criterion in the randomized SCOPE trial, and evidence on ACURATE neo in patients with bicuspid aortic stenosis is limited to a modestly sized case series. The performance of the ACURATE neo THV in bicuspid aortic stenosis was investigated in an international registry including 54 patients with a bicuspid aortic valve and 658 patients with a tricuspid aortic valve (Table 1) [17•]. Coronary occlusion occurred in one patient with a bicuspid valve (1.8%). After propensity score matching, a similar hemodynamic performance (9.8 ± 4.2 mmHg vs. 9.9 ± 4.5 mmHg, *P* = 0.944) and a comparable rate of relevant paravalvular regurgitation (7.4% vs. 5.5%, *P* = 0.734) were observed. However, the rate was significantly higher than that of unmatched tricuspid valve patients (7.4% vs. 3.1%, *P* = 0.0001), suggesting a substantially increased risk of paravalvular regurgitation. Thus, considering the rather low radial force of the ACURATE neo THV, it may not be an optimal device for often heavily calcified bicuspid aortic valves.

## The ACURATE neo for Degenerated Surgical Aortic Bioprostheses

The international experience of 85 patients using the ACURATE neo THV for the treatment of degenerated surgical aortic bioprostheses was reported from 14 centers in Europe and Canada (Table 1) [18•]. Malpositioning of the device was reported in two (2.4%) patients and coronary obstruction in one (1.2%) patient. Overall, an acceptable hemodynamic performance with an EOA of 1.5 ± 0.4 cm^2^ a mean gradient of 16 ± 8 mmHg was achieved despite the predominance of small-size (S) THVs. This study elucidated an important consideration for valve-in-valve procedures using the ACURATE neo THV. Since the upper crown is protruding 5 mm from the waist of the stent body, the crown may interfere with the stent posts and leaflets of the degenerated surgical aortic bioprosthesis, which may result in incomplete expansion, if the upper crown is positioned inside the bioprosthesis. In the study, a high implantation of the THV with the upper crown above the degenerated bioprosthesis was associated with significantly better hemodynamic outcomes (mean gradients: 14 ± 6 mmHg vs. 19 ± 10 mmHg, *P* = 0.001; EOA: 1.56 ± 0.37 cm^2^ vs. 1.36 ± 0.34 cm^2^, *P* = 0.045), but had increased risks of valve migration and early valve deterioration as compared with a low implantation. Further study is needed to clarify the short-term and long-term performance of this unique THV in the setting of valve-in-valve procedures for degenerated surgical aortic bioprostheses.

## The ACURATE neo THV for Native Pure Aortic Valve Regurgitation

TAVR is an alternative to SAVR for inoperable patients with aortic regurgitation. The off-label use of the ACURATE neo THV in patients with native pure aortic regurgitation is limited to small case series [19, 20]. Native pure aortic regurgitation is frequently characterized by the absence of annular and leaflet calcification, which complicates THV anchoring and increases the risk of device migration. The increased stroke volume secondary to aortic regurgitation and the presence of aortic root dilation render accurate device positioning difficult and may further increase the risk of device migration [24]. In this context, the ACURATE neo THV may have notable advantages owing to its unique x-shaped design and the deployment system (Fig. 1). In the absence of calcification, the protruding upper crown may serve as a safety anchor to prevent its dislocation into the left ventricular outflow tract (LVOT). Furthermore, the stable top-down two-step deployment system may be less affected by the increased stroke volume and facilitate the accurate positioning of the THV. Currently, two small multicenter experiences of the ACURATE neo THV for native pure aortic regurgitation have been published (Table 1) [19, 20]. In the first series of 20 patients, rapid pacing was used during THV implantation in 70% of the patients [19]. In one case (5%), a second THV implantation was required due to a low positioning of the initial THV. In contrast, in the second series of 24 cases, rapid pacing was used in 20% of cases only, and a second THV implantation was required in three cases (12.5%), in one case for severe paravalvular regurgitation (4.1%), and in two cases for device migration (8.3%) [20]. In the two case series, relevant paravalvular regurgitation was observed in one (5%) and two (8.3%) patients, respectively [19, 20]. Mean THV oversizing was close to 10% and resulted in new pacemaker implantation rates of 15% and 21%, respectively [19, 20]. In summary, transfemoral TAVR with the ACURATE neo THV may be an option for the treatment of pure aortic regurgitation in selected inoperable patients with suitable anatomy.

## Comparison with Other Contemporary Devices

Balloon-expandable THVs from the SAPIEN family and self-expanding THVs from the CoreValve/Evolut family have been extensively tested in randomized clinical trials and represent the benchmark for new devices [4]. The ACURATE neo THV has been compared to the balloon-expanding Edwards SAPIEN 3 THV in one randomized controlled trial and six propensity-score-matched studies [5•, 23–30] (Table 2).

**Table 2:** Results of comparison studies of ACURATE neo with SAPIEN 3 and Evolut R/PRO

Study design Author (year)	Baseline of ACURATE-arm			Valve performance		Clinical outcome		
	*N*	Age	Surgical score	Hemodynamic results	PVL	Pacemaker implantation	AKI stage 2 or 3	30-day mortality
*Versus SAPIEN 3*								
*PS-matched comparison* Husser et al. (2017) [25]	311	81 ± 6	LES: 18 ± 10%	9 ± 5 vs. 13 ± 5 mmHg (*P* < 0.001)	4.8% vs. 1.8% (*P* = 0.008)	10.2% vs. 16.4% (*P* = 0.018)	3.2% vs. 2.7% (*P* = 0.679)	2.3% vs. 1.9% (*P* = 0.742)
PS-matched comparison*^1^ Mauri et al. (2017) [23]	92	83 ± 7	LES: 16.2 ± 8.8%	9.3 ± 3.9 vs. 14.5 ± 5.5 mmHg (*P* < 0.001)	4.5% vs. 3.6% (*P* = 0.208)	12.0% vs. 15.2% (*P* = 0.678)	NA	1.1% vs. 2.2% (*P* = 1.000)
*PS-matched comparison* Schaefer et al. (2017) [26]	104	82 ± 6	LES: 15.9 ± 9.3% STS: 5.8 ± 3.8%	7.3 ± 2.8 vs. 11.8 ± 3.5 mmHg (*P* < 0.001)	4.8% vs. 1.9% (*P* = 0.257)	10.6% vs. 16.4% (*P* = 0.239)	2.9% vs. 1.9% (*P* = 0.655)	3.9% vs. 0.9% (*P* = 0.317)
PS-matched comparison*^2^ Husser et al. (2019) [27]	65	81 (77–84)	LES: 14.3% (9.8–21.5)	7(5–10) vs. 11(9–12, 13•, 14•) mmHg (*P* < 0.001)	4.6% vs. 0% (*P* = 0.244)	23.1% vs. 44.6% (*P* = 0.016)	1.5% vs. 7.7% (*P* = 0.208)	3.1% vs. 6.2% (*P* = 0.680)
*PS-matched comparison* Barth et al. (2019) [28]	329	81 ± 5	LES: 18.8 ± 14.7%	8.6 ± 4.6 vs. 10.9 ± 4.2 mmHg (*P* < 0.001)	12.0% vs. 3.1% (*P* < 0.001)	11.9% vs. 18.5% (*P* = 0.020)	NA	4.6% vs. 2.1% (*P* = 0.134)
*Randomized clinical trial+* Lanz et al. (2019) [5••]	372	83 ± 4	STS: 3.7% (2.5–4.9)	7(1–15) vs. 11(2–23) mmHg (*P* < 0.0001)	9.4% vs. 2.8% (*P* < 0.0001)	10% vs. 9% (*P* = 0.76)	3% vs. 1% (*P* = 0.0340)	2% vs. 1% (*P* = 0.09)
*Versus Evolut PRO*								
*PS-matched comparison* Pagnesi et al. (2019) [29•]	251	81 ± 7	ES II: 6.34 ± 5.21% STS: 5.08 ± 3.05%	8.3 ± 4.0 vs. 7.3 ± 3.6 mmHg (*P* = 0.003)	7.3% vs. 5.7% (*P* = 0.584)	11.0% vs. 12.8% (*P* = 0.565)	2.4% vs. 1.6% (*P* = 0.543)	3.2% vs. 1.2% (*P* = 0.221)
*Versus SAPIEN 3 versus Evolut R*								
*PS-matched comparison* Costa et al. (2020) [30]	48	82 (80–85)	STS: 4.0 ± 3.3%	8.4 ± 3.5 vs. 9.7 ± 7.5 vs. 6.1 ± 2.4 mmHg (*P* < 0.001)	0% vs. 0% vs. 2.1% (*P* < 0.01)	2.1% vs. 8.3% vs. 16.7% (*P* = 0.046)	1.0% vs. 2.2% vs. 2.7% (*P* = 0.659)	0% vs. 0% vs. 0% (*P* = NA)

*PS* propensity score, *PVL* paravalvular leak, *ES II* Euroscore II, *LES* logistic Euroscore, *STS* Society of Thoracic Surgeons predicted risk of mortality, *NA* not available/assessed

+ Independent event adjudication and echocardiographic core laboratory assessment were applied (no other studies comprised independent event adjudication or core laboratory echocardiographic assessment)

*^1^Selective cohort with an aortic annulus area < 400 mm^2^

*^2^Selective cohort with pre-existent right bundle branch block and no pacemaker at baseline

The SCOPE I trial was a randomized non-inferiority trial comparing ACURATE neo versus SAPIEN 3 in 739 patients with severe aortic stenosis with regard to a primary composite safety and efficacy endpoint at 30 days. TAVR with ACURATE neo failed to meet non-inferiority compared to SAPIEN 3 with respect to the composite of all-cause death, any stroke, life-threatening or disabling bleeding, major vascular complications, coronary artery obstruction, acute kidney injury (stage 2 or 3), rehospitalization for valve-related symptoms or congestive heart failure, valve-related dysfunction requiring repeat procedure, moderate or severe prosthetic valve regurgitation, or prosthetic valve stenosis within 30 days of the procedure [5••]. The difference was largely driven by a higher rate of moderate or severe paravalvular regurgitation in patients treated with ACURATE neo compared to SAPIEN 3 (9% vs. 3%, *P* < 0.0001). In turn, the transvalvular gradient was lower and the mean effective orifice area larger in patients treated with ACURATE neo compared with SAPIEN 3.

A low incidence of atrioventricular conductance disturbances requiring permanent pacemaker implantation compared not only to self-expanding but also to balloon-expandable devices has been considered an important strength of the unique design of the ACURATE neo THV. While retrospective cohort studies have shown a significantly lower rate of permanent pacemaker implantation in patients treated with ACURATE neo compared with SAPIEN 3 [24, 26, 27], findings from the SCOPE I trial indicate comparable rates of permanent pacemaker implantation (10% vs. 9%, *P* = 0.76) [5••]. Interestingly, a multicenter study including only patients with pre-existing right bundle branch block demonstrated a significantly lower incidence of new pacemaker implantation in the ACURATE neo THV as compared to the SAPIEN 3 THV (23.1% vs. 44.6%, *P* = 0.016) [26]. The SCOPE I trial also suggested an increased risk of acute kidney injury following TAVR in the ACURATE neo THV, which may be attributable to increased contrast volume associated with the longer procedural time due to more frequent pre- and post-dilation [5••]. Although the difference was not observed in other retrospective studies, rates of pre- and post-dilation were consistently higher in the ACURATE neo THV [23–27], and some studies showed larger contrast volume and longer procedural time as compared to the SAPIEN 3 THV [25, 28].

A randomized clinical trial comparing the ACURATE neo THV versus the Evolut R/PRO THV has recently completed patient enrollment, and the results will soon be available (NCT03192813). A propensity-score-matched comparison suggested comparable short-term outcomes, including device success, relevant paravalvular regurgitation, all-cause death, stroke, new permanent pacemaker, and early safety endpoint, between the two self-expanding THVs (Table 2) [29•]. Data on long-term valve durability is missing at this stage.

## Place for the ACURATE neo THV

The ACURATE neo THV may not be the optimal device for patients with excessive leaflet calcification and LVOT calcification, in which an increased risk of paravalvular regurgitation following TAVR is anticipated [31–34]. However, some specific patient groups may benefit from the unique design of the ACURATE neo THV.

### Patients at High Risk of Conduction Disturbances

A low risk of permanent pacemaker implantation compared with other self-expanding devices makes ACURATE neo a valuable device in patients with pre-existing conduction disturbances, i.e., right bundle branch block, and may facilitate early discharge following TAVR [27, 35].

### Patients at High Risk of Coronary Obstruction or Future Coronary Re-access

Conceptually, the THV may mitigate the risk of coronary obstruction in patients with low coronary distance, small sinus of Valsalva, and long leaflets [36, 37]. Furthermore, coronary re-access may be less challenging as compared to the other THVs because of the short stent body and the open-cell design of the upper crown. Patients with coronary artery disease at risk of future percutaneous coronary interventions may be suitable candidates for this THV [38–40].

### Patients at High Risk of Prosthesis-Patient Mismatch

The ACURATE neo valve may be useful in patients with a small anatomy that my benefit from the supra-annular design to reduce the risk of prosthesis-patient mismatch [22].

### Patients with Horizontal Aorta

Increased aortic root angulation has been shown to adversely influence acute procedural success following other self-expanding THV (CoreValve) possibly due to its long stent frame and less flexible delivery system [41]. Therefore, the short stent frame and the flexible delivery system of the ACURATE neo may be advantageous in these situations. The unique stabilization arches may also provide a better co-axial alignment and facilitate accurate deployment.

## Conclusions

The ACURATE neo THV conceptually differs from other self-expanding valves with a two-step top-down release mechanism. Low radial strength reduces the need for permanent pacemaker implantation and comes at the expense of an increased risk of paravalvular aortic regurgitation compared to the SAPIEN 3 THV. Data on long-term outcomes and valve durability is missing.
